# Experimental Study on Rheological Behavior of Firefighting Foams

**DOI:** 10.3390/ma18143236

**Published:** 2025-07-09

**Authors:** Youquan Bao, Huiqiang Zhi, Lu Wang, Yakun Fan, Junqi Wang

**Affiliations:** 1 Tianjin Key Laboratory of Fire Safety Technology, Key Laboratory of Fire Protection Technology for Industry and Public Building of Ministry of Emergency Management, Tianjin Fire Science and Technology Research Institute of MEM, Tianjin 300381, China; 2 Jiangsu Suolong Fire Science and Technology Co., Ltd., Taizhou 225753, China

**Keywords:** firefighting foam, foam rheology, foam aging, foam coarsening, elasto-viscoplastic, yielding

## Abstract

The rheological behavior of firefighting foam is the basis for analyzing foam flow and foam spreading. This experimental study investigates the complex rheological behavior of rapidly aging firefighting foams, specifically focusing on alcohol-resistant aqueous film-forming foam. The primary objective is to characterize the time-dependent viscoelasticity, yielding, and viscous flow of firefighting foam under controlled shear conditions, addressing the significant challenge posed by its rapid structural evolution (drainage and coarsening) during measurement. Using a cylindrical Couette rheometer, conductivity measurements for the liquid fraction, and microscopy for the bubble size analysis, the study quantifies how foam aging impacts key rheological parameters. The results show that the creep and relaxation response of the firefighting foam in the linear viscoelastic region conforms to the Burgers model. The firefighting foam shows ductile yielding and significant shear thinning, and its flow curve under slow shear can be well represented by the Herschel–Bulkley model. Foam drainage and coarsening have competitive effects on the rheology of the firefighting foam, which results in monotonic and nonmonotonic variations in the rheological response in the linear and nonlinear viscoelastic regions, respectively. The work reveals that established empirical relationships between rheology, liquid fraction, and bubble size for general aqueous foams are inadequate for firefighting foams, highlighting the need for foam-specific constitutive models.

## 1. Introduction

Firefighting foam is the most common and practicable method for the fire protection of flammable liquids and is widely used in places where flammable liquids are produced, processed, stored, and used [1,2,3], such as oil depots, petrochemical platforms, coal chemical enterprises, and airports. In the case of fire caused by flammable liquid, firefighting foam is applied to the protected area and spreads over the liquid surface to form a foam blanket [4,5]. The foam blanket can resist the radiation of flame to flammable liquid, inhibit flammable liquid evaporation, and block the contact of flammable liquid vapor with air to achieve the purpose of fire control and extinguishing [6]. The driving force of the foam spread is due to differences in the hydrostatic pressure within the firefighting foam, whereas the dominating resisting force is due to viscous friction between the foam blanket and the flammable liquid. The friction resistance is closely related to the non-Newtonian rheological behavior of firefighting foam, and quantifying rheological properties is fundamental for modeling foam spread dynamics [7,8]. Understanding time-dependent rheological behavior is particularly crucial for rapidly aging firefighting foams, as their structural evolution impacts performance in real-world applications.

The applicable scenarios of firefighting foams generated by different foam concentrates vary significantly. Aqueous film-forming foam (AFFF) is widely recognized for its film-forming capability on flammable liquid surfaces and commendable fire suppression efficiency [9,10]. However, due to the environmental hazards posed by perfluorooctane sulfonate (PFOS) and its derivatives [11], the use of AFFF is restricted in environmentally sensitive areas (e.g., ecological reserves, drinking water sources, permanent basic farmland) and faces challenges in formula modification [12,13]. In these sensitive zones, fluoroprotein foam (FP) serves as a viable alternative. When protecting polar flammable liquids, alcohol-resistant foams such as alcohol-resistant aqueous film-forming foam (AFFF/AR) or alcohol-resistant fluoroprotein foam (FP/AR) must be employed [3]. Alcohol-resistant foam concentrates are typically formulated by incorporating high-molecular-weight polysaccharides like xanthan gum into common foam concentrates, or by appropriately increasing the concentration of fluorocarbon surfactants. These modifications can partially mitigate the dehydration effect of polar liquids on firefighting foams, though their selection must still comply with environmental protection regulations. The development of fluorine-free foam concentrate appears to be an emerging trend, though current engineering applications remain limited. A study funded by the National Fire Protection Association reveals substantial variations in the properties of fluorine-free foams currently developed by different manufacturers [14]. In terms of current engineering applications, particularly in China, AFFF and FP foams, including alcohol-resistant variants, dominate in terms of usage proportion, while fluorine-free foams remain predominantly in the research and development phase.

Firefighting foam is aqueous foam, a dispersion of a gas into a surfactant aqueous solution. From a fluid mechanics perspective, aqueous foam behaves as a complex soft material whose stress response incorporates elastic, viscous, and plastic components [15,16]. Aqueous foam exhibits the hallmark of non-Newtonian characteristics including shear-thinning viscosity, yield stress, and viscoelasticity, rendering classical Newtonian fluid mechanics inadequate. A rheological analysis provides the framework to quantify these complex behaviors through constitutive modeling [17]. Since the Nobel Prize winner Pierre-Gilles de Gennes proposed the concept of “soft matter” in 1991, with the development of soft matter rheology, scholars have carried out considerable research on the rheological behavior of aqueous foam. The related research has focused mainly on the mechanism and suppression of aqueous foam wall slip, the experimental methods of aqueous foam rheology, the factors affecting the rheological behavior of aqueous foam, and the development of constitutive models of aqueous foam [18]. At present, the mechanism of aqueous foam sliding on a solid surface has been determined, and methods for restraining wall slip in the rheological measurement of aqueous foam have been developed [19]. On the mesoscopic scale, aqueous foam aging is mainly manifested as follows: the continuous liquid phase flows downward along the bubble gap under the effect of gravity, resulting in a decrease in the local liquid fraction, that is, foam drainage occurs [20,21]; the dispersed gas phase diffuses under the effect of the pressure difference between bubbles, resulting in a decrease in the number of bubbles and an increase in the average bubble size, which is referred to as foam coarsening [22,23]. The non-Newtonian rheological behavior of aqueous foam is closely related to the foam liquid fraction and bubble size [24]. Quantitative relationships between rheological parameters such as the shear modulus, yield stress, and consistency coefficient of aqueous foam and the liquid fraction, bubble size, and liquid surface tension have been established [25,26,27].

However, the existing experimental studies on the rheological behavior of aqueous foam are almost all aimed at foams with relatively stable structures (slow aging process), and relevant models and quantitative relationships have also been established on the basis of these experimental studies. Firefighting foam shows remarkable foam drainage and foam coarsening [28,29]. The quarter drain time of commercially available firefighting foam is usually several minutes, and such rapid structural changes make it difficult to measure rheological properties. According to the literature, in the middle of the last century, scholars attempted to measure the viscosity of firefighting foam. There is no doubt that these studies are groundbreaking, but from the perspective of rheology, these measurement processes have no clear shear conditions and can only measure the viscosity of firefighting foam at a certain moment in the entire life of the foam. Elliott et al. [30] were the first to perform continuous rheological measurements of firefighting foams and measured the yield stress of various firefighting foams. Gardiner et al. [31] built a Poiseuille flow rheometer and analyzed the wall slip of compressed air firefighting foam in a pipe flow. Gao et al. [15] continuously measured the apparent viscosity of 3% aqueous film-forming firefighting foam and tried to correlate the rheological curve of firefighting foam with the liquid fraction. In general, research on the rheological behavior of firefighting foam is not very common.

To enhance the understanding of the rheological behavior of firefighting foam, in the present work, the viscoelasticity, yielding, and viscous flow of firefighting foam were experimentally studied, and the effects of foam aging on rheological behavior were analyzed. In Section 2, the foam concentrate, firefighting foam production and experimental protocol are introduced. In Section 3, the rheological response of firefighting foam under different loading modes is displayed, and the effect of foam aging on the rheological response is analyzed. Finally, the main conclusions are summarized in Section 4.

## 2. Materials and Methods

### 2.1. Foam Concentrate and Firefighting Foam Production

An alcohol-resistant aqueous film-forming foam concentrate (3% AFFF/AR) provided by Jiangsu Suolong Fire Science and Technology Co., Ltd. (Taizhou, China) was used as the foaming agent. The foam concentrate contained 25 wt.% cocamidopropyl betaine, 6 wt.% alkyl glycosides, 4 wt.% fluorocarbon surfactants, and 1 wt.% xanthan gum, with a density of 1030 kg/m^3^ and a pour point of −40 °C. The foam concentrate was mixed with water at a volume ratio of 3:97 to form a foam solution with a surface tension of 18.7 mN/m. Firefighting foam was prepared by whipping air into 110 mL of the above solution using a mixer (HS-U-120W, Zhengzhou Dejing Instrument Equipment Co., Ltd., Zhengzhou, China) equipped with a three-blade propeller for 4 min at 2000 rpm. The expansion ratio of the firefighting foam prepared by this method was approximately 7.2.

### 2.2. Experimental Protocol

Firefighting foam is subjected to drainage and coarsening once it is generated. To analyze the rheology of firefighting foam quantitatively, it is necessary to measure the liquid fraction and bubble size in addition to the rheological response.

The vertical profile of the local liquid fraction in the firefighting foam was measured by electrical conductivity. A conductivity meter (DDSJ-307F, INESA Scientific Instrument Co., Ltd., Shanghai, China) with a range of 0.001 μS/cm~1000 mS/cm and a resolution of 0.001 μS/cm was used to measure the conductivity of the firefighting foam *ψ*_foam_ and foam solution *ψ*_solution_. By adjusting the electrode position of the conductivity meter and repeating the test, the conductivity of the firefighting foam at different vertical positions was obtained. According to the relationship between the relative conductivity, *ψ* = *ψ*_foam_/*ψ*_solution_, and the liquid fraction *ϕ_l_* of aqueous foam shown in Equation (1) [32], the vertical profile and time evolution of the local liquid fraction in the firefighting foam were estimated.

(1) $$\phi_{l}=\frac{3\psi (1+11\psi )}{1+25\psi +10\psi^{2}}$$

**Figure 1:**
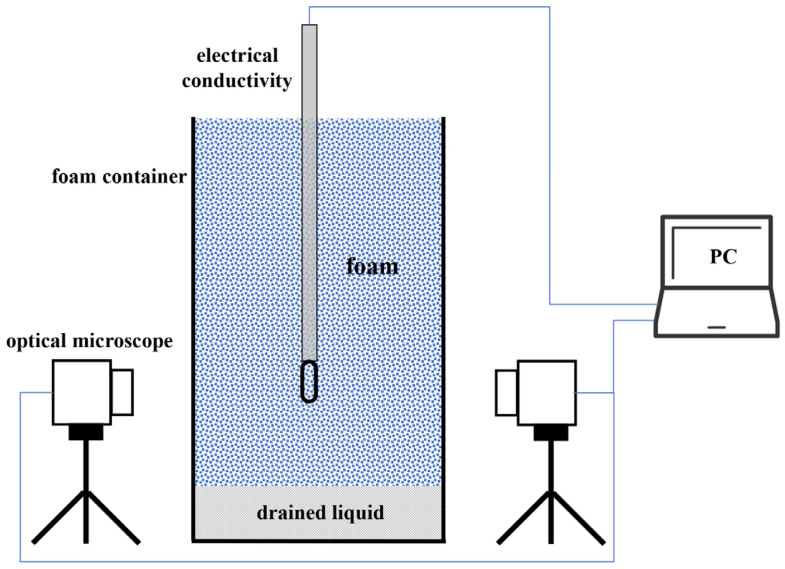
Schematic diagram of foam conductivity and bubble size test.

The bubble size of the firefighting foam was measured by using optical microscopy. Three autofocusing microscopes (3R-MSBTVTY, 3R Group. 3R SOLUTION Corp., Tokyo, Japan) were used to take photomicrographs of the firefighting foam through the transparent wall of the foam container. The magnification of the microscope could be manually switched between 40×, 100×, 180× and 250×. By adjusting the position of the microscope and repeating the test, photomicrographs of the firefighting foam at different vertical positions were obtained. ImageJ (v1.54f, National Institute of Health, Bethesda, MD, USA), a freely downloadable image analysis software package, was used to statistically analyze firefighting foam photomicrographs, which included the number and Feret diameter of bubbles. The experimental setup for measuring foam conductivity and bubble size is illustrated in Figure 1.

All rheological measurements were performed with a commercial rheometer (MCR92, Anton Paar, Graz, Austria). In previous rheological experiments of aqueous foams, cone-plate, parallel plate, and cylindrical Couette systems have all been successfully used as shear geometries [33]. However, considering the remarkable drainage of firefighting foam, when a cone-plate or parallel plate system is adopted, then the drained liquid accumulates at the bottom plate, which inevitably affects the measurement results. Therefore, the cylindrical Couette system is considered more suitable for rheological measurements of firefighting foam. The B-CC39/P6 inner cylinder (outer diameter of 40 mm, height of 60 mm) was paired with a custom-made outer cylinder (inner diameter of 90 mm, height of 140 mm) fixed with an Anton Paar flexible cup holder to form a cylindrical Couette system. The surfaces of the inner cylinder and outer cylinder were scored to prevent wall slip. Notably, there should be sufficient space between the bottom of the inner cylinder and the bottom of the outer cylinder, 3 cm in the present study, to contain the drained liquid. In other words, the inner cylinders were immersed in firefighting foam throughout the rheological tests.

There exists an inevitable time delay between the completion of firefighting foam preparation and the commencement of measurements. In the present study, this time delay was controlled within 30 s. To standardize experimental conditions, the moment of foam preparation completion was designated as time zero (*t* = 0 min), with all subsequent measurements being initiated at the 0.5 min mark.

## 3. Results and Discussion

### 3.1. Foam Aging

**Figure 2:**
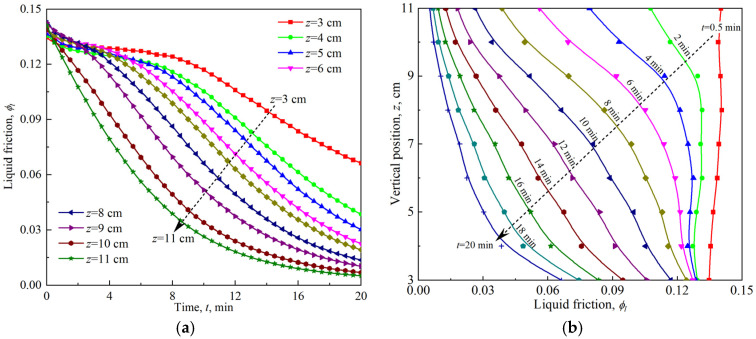
Free drainage of firefighting foam: (**a**) time evolution of the local liquid fraction at different vertical positions; (**b**) vertical profile of the local liquid fraction as a function of time.

Figure 2 shows the time evolution and vertical profile of the local liquid fraction measured by electrical conductivity in firefighting foam with a height of 11 cm. At *t* = 0.5 min, the initial local liquid fraction is almost uniform and constant, *ϕ_l_*_,0_ ≈ 0.14, which is undoubtedly equal to the reciprocal of the foam expansion ratio. Over time, the liquid drains from the firefighting foam, flows downward, and accumulates at the base of the container. The entire drainage process can be roughly divided into two stages. For approximately *t* < 10 min, the liquid fraction at the upper part of the foam decreases, the drainage front, referred to as the lower limit of this region, moves downward, and the width of the front increases. The lower part of the foam undergoes steady drainage, with an almost constant liquid fraction. At approximately *t* > 10 min, the drainage front reaches the bottom of the foam. The foam at different vertical positions continues to drain, becomes drier, and gradually develops toward the equilibrium profile.

**Figure 3:**
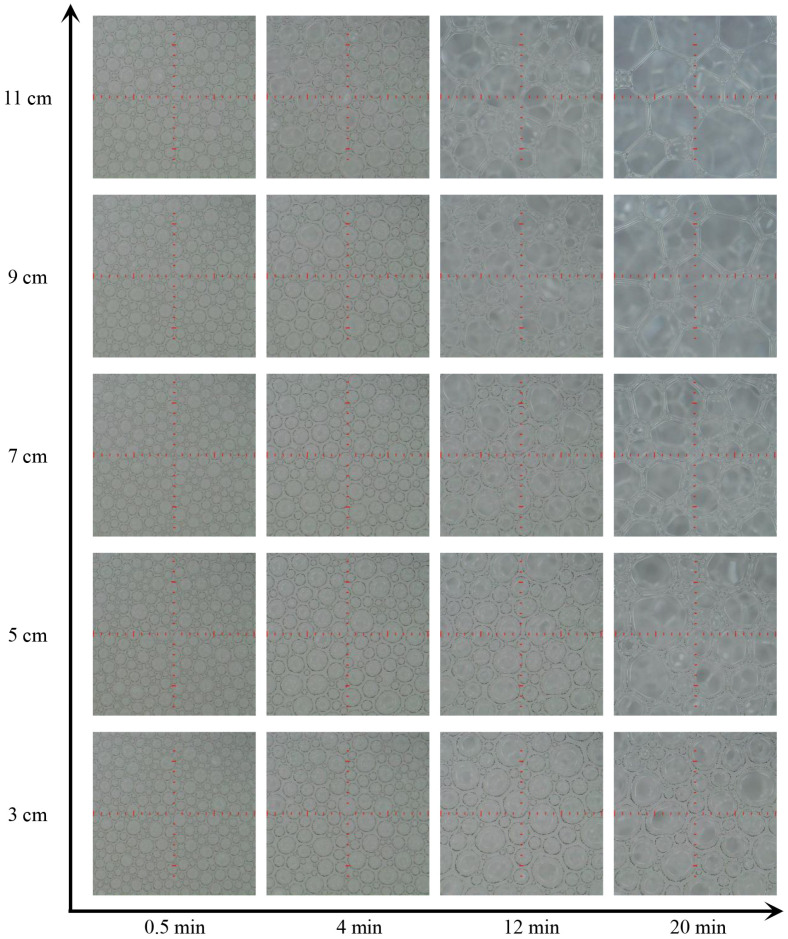
Photographs at different vertical positions in the firefighting foam at *t* = 0.5 min, 4 min, 12 min, and 20 min.

**Figure 4:**
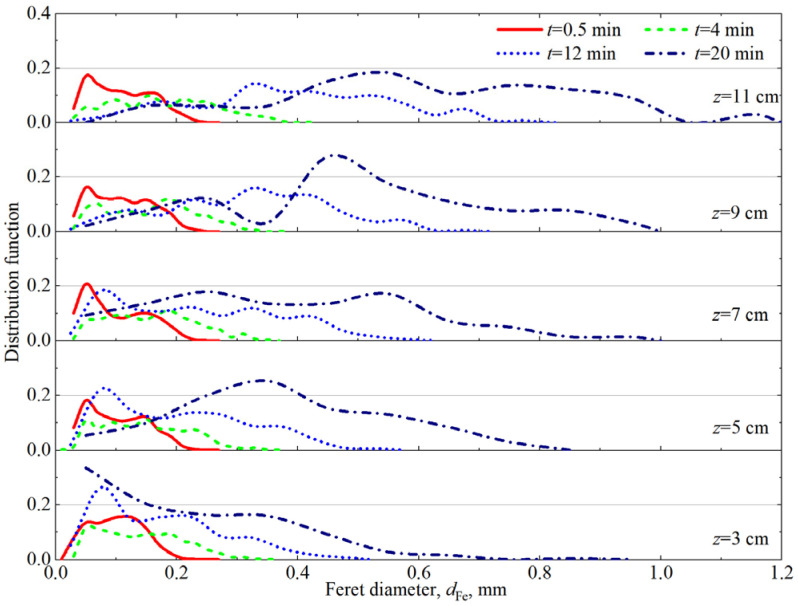
Bubble size distributions at different vertical positions in the firefighting foam.

In Figure 3, photographs at different vertical positions in the firefighting foam show the shapes and sizes of the bubbles at *t* = 0.5 min, 4 min, 12 min, and 20 min. All foams at different vertical positions contain wet spherical bubbles at the start of imaging, *t* = 0.5 min, and over time, the number of these bubbles decreases, and the bubble becomes larger and more polyhedral. In Figure 4, the bubble size distributions at different vertical positions in the firefighting foam become more discrete over time, and the bubble sizes in the upper part of the foam are more discrete than those in the lower part of the foam at a given time. The evolution of bubble size distributions is complex and difficult to quantify, and a common strategy is to analyze the average bubble size. Various average sizes, such as the arithmetic mean radius *R*_A_ = <*r*>, the mean square radius *R*_rms_ = <*r*^2^>^1/2^, the cubic mean radius *R*_o_ = <*r*^3^>^1/3^, and the Sauter mean radius *R*_32_ = <*r*^3^>/<*r*^2^>, have been used in previous studies, but it is still not clear which average method is most appropriate for bubble size statistical analysis [28]. In conjunction with the theoretical analysis of firefighting foam rheology, the time evolution of *R*_A_ and *R*_32_ was calculated, as presented in Table 1, and was used in subsequent foam rheology analysis. *R*_A_ and *R*_32_ of the bubble increased over time, and the mean bubble radius in the upper part of the foam was larger than that in the lower part. *R*_32_ was larger than *R*_A_, and the ratio *R*_32_/*R*_A_ ≈ 1.3~1.9.

### 3.2. Viscoelasticity of Firefighting Foam

**Figure 5:**
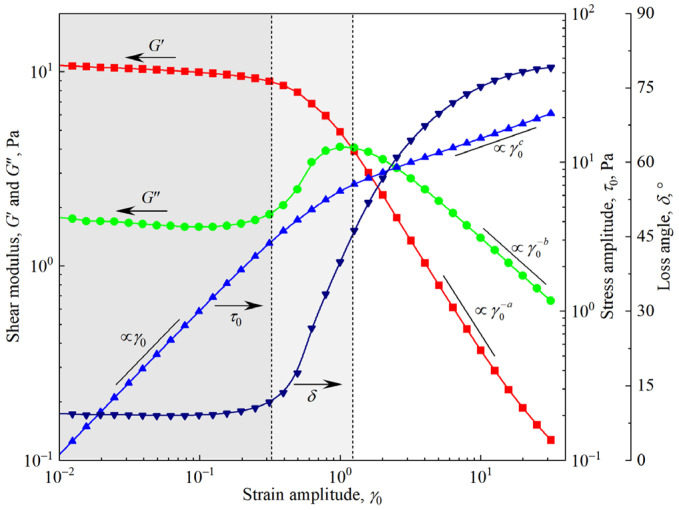
Typical results of the strain amplitude sweep test for firefighting foam (*f* = 1 Hz).

Figure 5 shows the typical response of the stress amplitude *τ*_0_, shear moduli *G*′ and *G*″, and loss angle *δ* of the firefighting foam in the strain amplitude *γ*_0_ sweep test. There are three distinct regions: the linear region, transition region, and nonlinear region. In the linear region, the storage modulus *G*′ is greater than the loss modulus *G*″, and *δ* is much smaller than 45°. *G*′, *G*″, and *δ* are almost independent of *γ*_0_, and *τ*_0_ is proportional to *γ*_0_ ($\tau_{0}\propto \gamma_{0}$). In the nonlinear region, *G*″ is greater than *G*′, and *δ* is greater than 45°. Both *G*′ and *G*″ decrease with a power law ($G^{\prime }\propto \gamma_{0}^{-a},G^{\prime \prime }\propto \gamma_{0}^{-b},a>b$), whereas *τ*_0_ increases with a power law ($\tau_{0}\propto \gamma_{0}^{c},c<1$). The transition region is between the linear region and the nonlinear region. In the transition region, *G*′ decreases monotonically, whereas *G*″ increases first and then decreases, and the foam yields. The stress amplitude curves in the linear and nonlinear regions are extended, and the intersection point falls in the transition region; that is, the yield point and the corresponding stress and strain are the yield stress *τ*_y_ and yield strain *γ*_y_, respectively.

**Figure 6:**
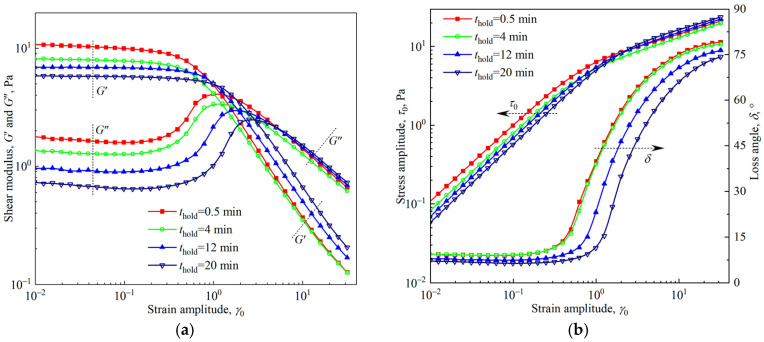
Strain amplitude sweep tests for the firefighting foams after different holding times: (**a**) shear modulus vs. strain amplitude; (**b**) stress amplitude and loss angle vs. strain amplitude.

**Figure 7:**
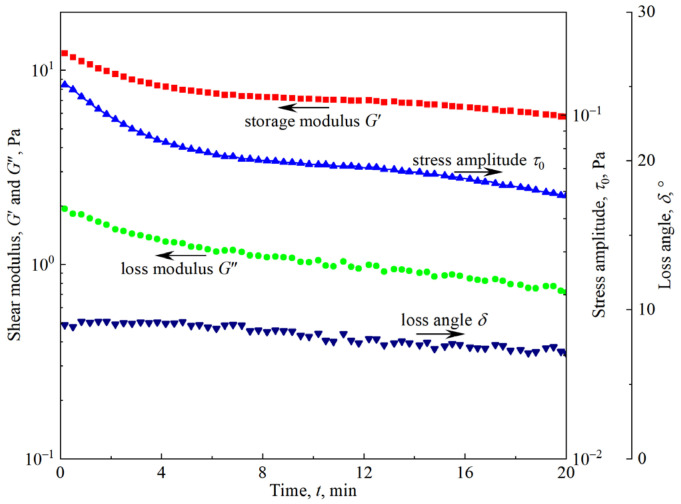
Time sweep test within the linear viscoelastic region of the firefighting foam (*γ*_0_ = 0.01, *f* = 1 Hz).

To explore the effect of foam aging on viscoelasticity, strain amplitude sweep tests were carried out after the firefighting foam was held for *t*_hold_ = 0.5 min, 4 min, 8 min, 12 min, 16 min, and 20 min. To ensure clarity, only partial test results are shown in Figure 6. An attempt to unify the curves under different holding times onto a single curve by normalizing the coordinate axis of Figure 6a using *G*′ in the linear region and *γ*_y_ did not succeed. The reason was that foam aging had different effects on the linear region and the nonlinear region. Figure 6 shows that in the linear region, the effect of foam aging is monotonic, and both the shear modulus and stress amplitude decrease with holding time. The time sweep test within the linear viscoelastic region (*γ*_0_ = 0.01, *f* = 1 Hz) shown in Figure 7 also confirms this monotonic aging effect. In the nonlinear region, the power law exponents *a*, *b*, and *c* are almost independent of the holding time *t*_hold_, where *a* ≈ 1.1, *b* ≈ 0.63, and *c* ≈ 0.35. However, the curves corresponding to *t*_hold_ = 4 min appear at the bottom of the curve cluster, indicating that the effect of foam aging is nonmonotonic. This effect cannot be attributed to test bias, as identical nonmonotonic aging behavior is replicated in the test results of Section 3.4.

**Figure 8:**
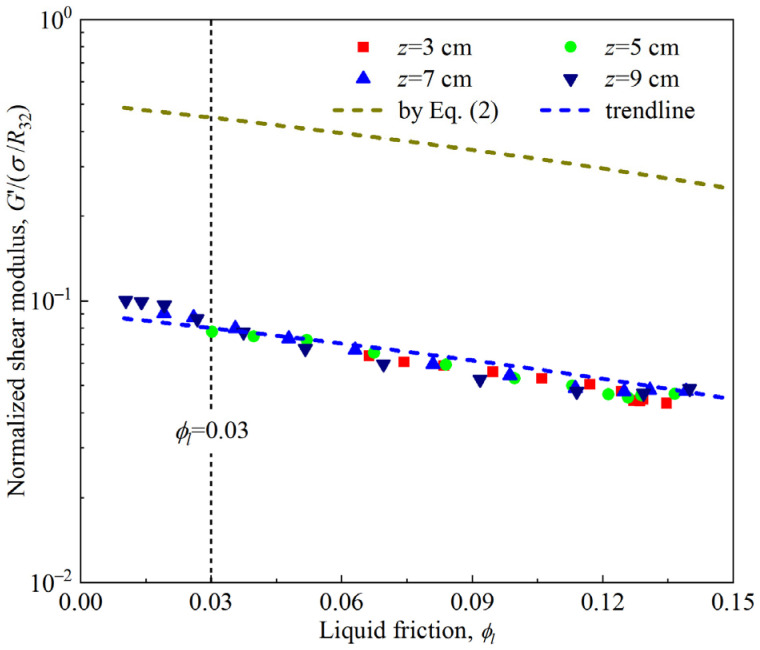
Dependence of the normalized storage modulus on the liquid fraction at different vertical positions in the firefighting foam.

Numerous experimental results have shown that, for polydisperse aqueous foam, the variation in the shear modulus in the linear region with the liquid friction *ϕ_l_* and Sauter mean radius *R*_32_ can be well described by the empirical relation shown in Equation (2) [18,27,34]. The applicability of this empirical relation to firefighting foam was examined herein. Notably, the rheological response of the firefighting foam was measured with a cylindrical Couette system with an inner cylinder height of 60 mm in this work. At this height span, the evolutions of *ϕ_l_* and *R*_32_ of the foams are different, as shown in Figure 2 and Table 1. A correlation analysis was conducted between the *ϕ_l_* and *R*_32_ of the foam at *z* = 3 cm, 5 cm, 7 cm, and 9 cm and the rheological response. In Figure 8, the normalized storage modulus shows good consistency; it is almost independent of the vertical positions of the foam but less than the calculation result of Equation (2). If the factor 1.4 in Equation (2) is changed to 0.2, then, as shown in the trend line in Figure 8, the test results are consistent with the relationship, but this treatment is a complete mathematical fit and lacks physical support.

(2) $$G=1.4(1-\phi_{l})(\phi_{l}^{*}-\phi_{l})\frac{\sigma }{R_{32}}\;\;\;\;\;\;(0.03<\phi_{l}<0.36)$$

**Figure 9:**
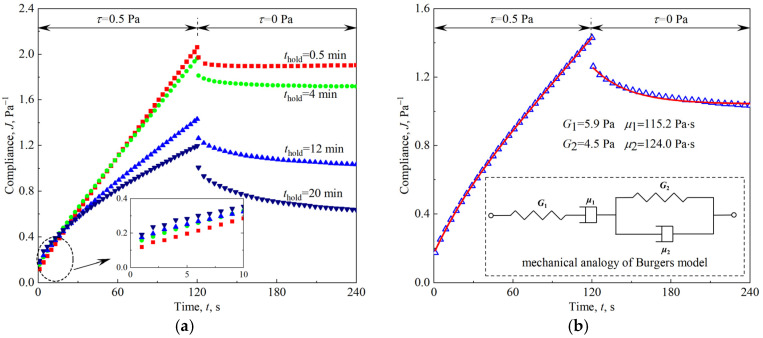
Creep tests for firefighting foams: (**a**) after different holding times; (**b**) fitting and prediction of the Burgers model.

Creep tests were also carried out to analyze the deformation response of the firefighting foam after loading and unloading stress in the linear region. The loaded creep stress was 0.5 Pa, and both the creep and relaxation processes lasted for 2 min. As shown in Figure 9a, transient deformation and continuous deformation occurred after the firefighting foam was loaded with stress, and a transient rebound, delayed recovery, and residual deformation occurred after unloading. These deformation responses conformed to the Burgers model, whose mechanical analogy and constitutive model are shown in Figure 9b and Equation (3) [35]. The Burgers model’s mechanical analogy comprises a Maxwell element (spring *G*_1_ in series with dashpot *μ*_1_) connected in series with a Kelvin–Voigt element (spring *G*_2_ parallel to dashpot *μ*_2_). This configuration captures instantaneous elastic deformation (*G*_1_), retarded viscoelastic relaxation (*G*_2_ and *μ*_2_), and viscous flow (*μ*_1_), providing a physically interpretable framework for foam viscoelasticity. The creep curve was used to fit the parameters of the Burgers model, the relaxation curve was predicted by the determined parameters, and both the fitting and prediction results were satisfactory, as shown in Figure 9b. The parameters of the Burgers model for the creep tests of the firefighting foams with different holding times are shown in Table 2, and all the parameters change monotonically.

(3) $$\left\{\begin{array}{c} J=\frac{1}{G_{1}}+\frac{1}{G_{2}}[1-exp(-\frac{G_{2}}{\mu_{2}}t)]+\frac{1}{\mu_{1}}t\;\;\;\;\;\;(creep)\\ J=J_{\infty }+\frac{1}{G_{2}}exp(-\frac{G_{2}}{\mu_{2}}t)\;\;\;\;\;\;(relaxation) \end{array}\right.$$

### 3.3. Yielding of the Firefighting Foam

**Figure 10:**
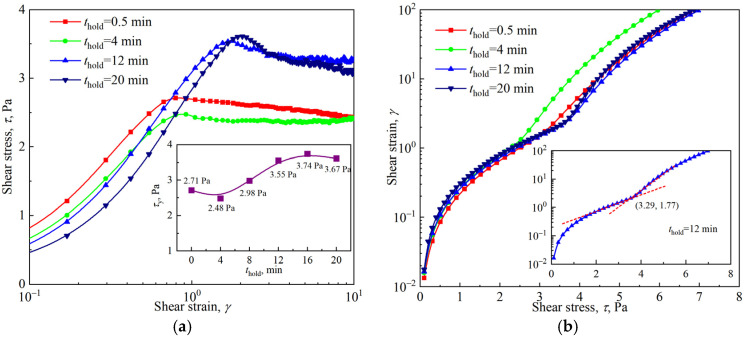
Yielding of the firefighting foams: (**a**) start-up flow at a constant shear rate of 0.125 s^−1^; (**b**) stress sweep test.

**Figure 11:**
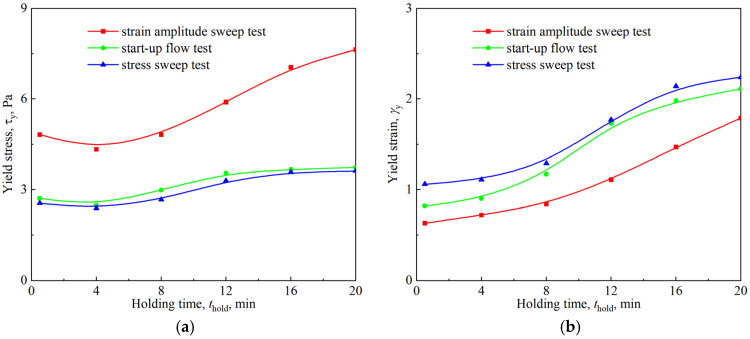
Yield stress and yield strain of the firefighting foams measured by using different methods: (**a**) yield stress vs. holding time; (**b**) yield strain vs. holding time.

In addition to the strain amplitude sweep tests shown in Figure 6, start-up flow tests and stress sweep tests were also carried out to determine the yield stress and yield strain of the firefighting foams. As shown in Figure 10a, the stress and strain corresponding to the highest point in the start-up flow test are the yield stress and yield strain, provided that the applied shear rate is small enough; it was 0.125 s^−1^ in this study. The determination of yield stress and yield strain in the stress sweep test also required an extension of the curve, as shown in Figure 10b, just as in the strain amplitude sweep test. The yield stress and yield strain measured by the three methods are shown in Figure 11.

**Figure 12:**
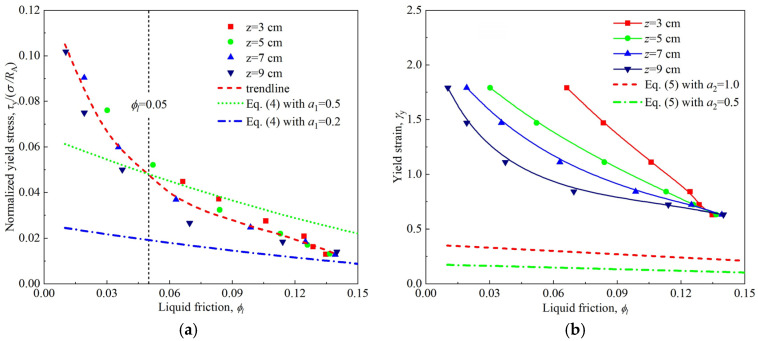
Dependence of the normalized yield stress and yield strain on the liquid fraction at different vertical positions in the firefighting foam: (**a**) normalized yield stress vs. liquid fraction; (**b**) yield strain vs. liquid fraction.

Unlike classic brittle fracture yielding, the yield process of firefighting foam was relatively smooth and involved ductile yielding, as shown in Figure 10b. The results of the three test methods showed that with the foam aging, the yield strain increased monotonically, whereas the yield stress decreased slightly at first and then increased. The difference was that the yield points measured by the start-up flow and stress sweep were similar, whereas the yield stress obtained by the strain amplitude sweep was larger, and its yield strain was smaller. This inconsistency of yield values caused by different test methods, especially those involving oscillatory shear and simple shear, is a common situation in soft-material rheological measurements [36]. Exploring this general problem of rheological measurement was not the objective of this study. Previous studies have shown that the dependence of the yield stress and yield strain measured in the oscillatory mode on the bubble size, surface tension, and liquid fraction in aqueous foams can be described by the following empirical expressions [18]:

(4) $$\tau_{y}={a_{1}\frac{\sigma }{R_{A}}(\phi_{l}^{*}-\phi_{l})}^{2}\;\;\;\;\;\;(0.05<\phi_{l}<0.25)$$

(5) $$\gamma_{y}=a_{2}(\phi_{l}^{*}-\phi_{l})$$

where *a*_1_ is a constant between 0.2 and 0.5, and *a*_2_ is a constant between 0.5 and 1. When combined with Figure 2a, Table 1, and Figure 11, the intermediate variable of time was eliminated, and the dependence of the normalized yield stress and yield strain on the liquid fraction could be obtained, as shown in Figure 12. The variation in the normalized yield stress with the liquid fraction of the firefighting foam seemed to fall on a trend line, which was independent of the vertical position of the firefighting foam. Within the scope of application, that is, 0.05 < *ϕ_l_* < 0.25, Equation (4) was used to characterize the change in normalized yield stress with the liquid fraction of the firefighting foam, where *a*_1_ was approximately 0.38. However, the variation in yield strain with the liquid fraction at different vertical positions in the firefighting foam did not return to a trend line and far exceeded the trend and interval predicted by Equation (5).

### 3.4. Viscous Flow of Firefighting Foam Under Slow Shear

**Figure 13:**
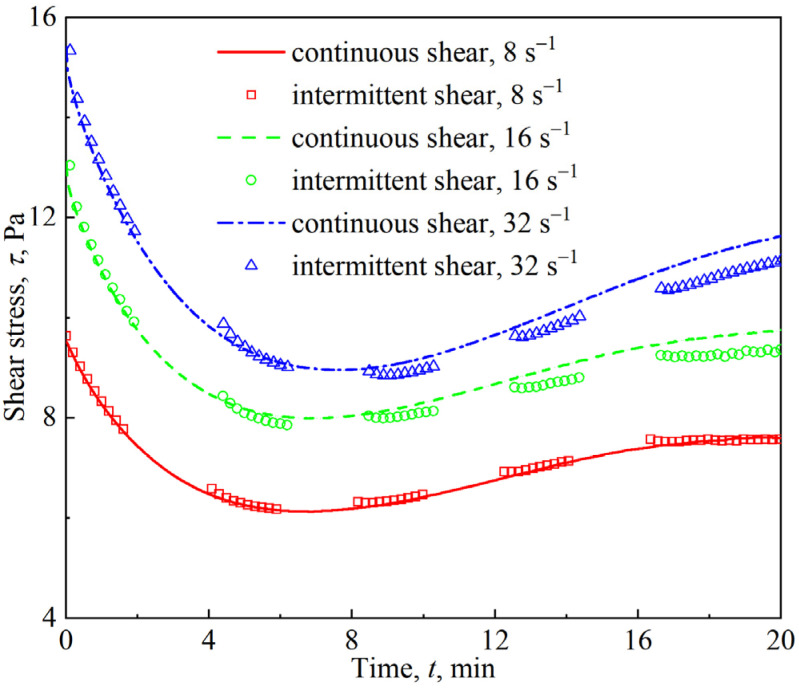
Stress response of firefighting foam under continuous and intermittent shear conditions of 8 s^−1^, 16 s^−1^, and 32 s^−1^.

Viscous flow occurs after the firefighting foam yields. To measure the apparent viscosity of the firefighting foam, shear must be applied. High continuous shear inevitably affects foam drainage and coarsening, and the analysis of this coupling is difficult at present. This study focused on the viscous flow of firefighting foam under slow shear, in which the effect of shear on foam drainage and coarsening is negligible. As shown in Figure 13, continuous and intermittent shear at different constant shear rates led to differences in shear stress evolution over time. It seemed that the shear rate of 8 s^−1^ was tolerable. The response results of continuous and intermittent shear at shear rates above 16 s^−1^ were significantly different, especially in the latter part of the shear tests. Therefore, the shear rate of the viscous flow of the firefighting foam in this work was controlled within 8 s^−1^.

**Figure 14:**
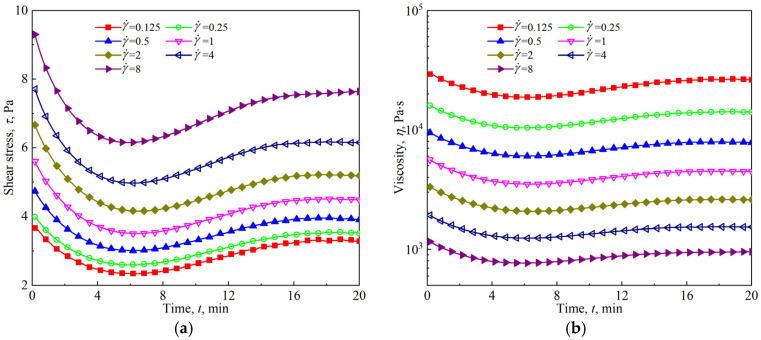
Constant shear rate tests for firefighting foams: (**a**) time evolution of shear stress; (**b**) time evolution of viscosity.

**Figure 15:**
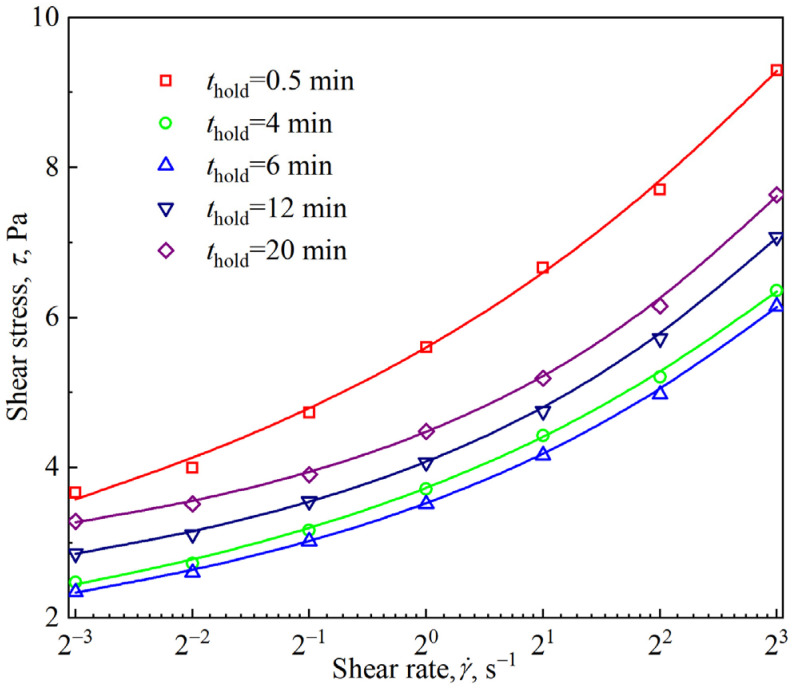
Flow curves of the firefighting foams (the symbols represent the measured data, and the solid lines represent the results fitted by the Herschel–Bulkley model).

**Figure 16:**
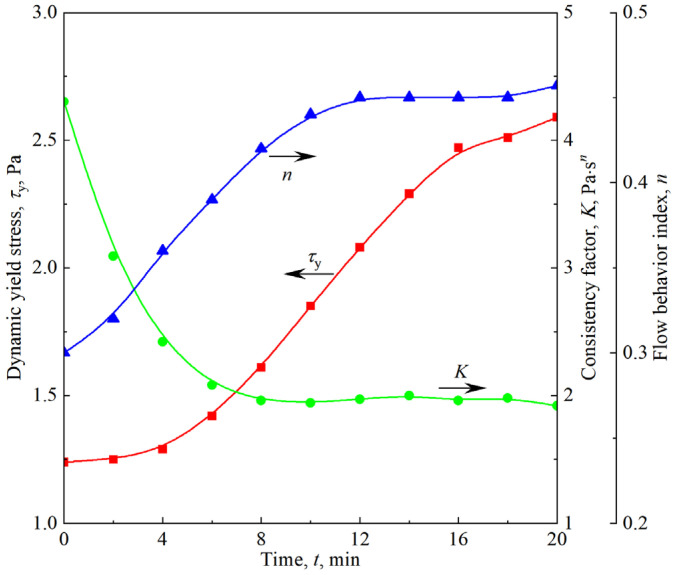
Evolution of the fitted parameters of the Herschel–Bulkley model with aging time.

Figure 14 shows the time evolution of the stress response and viscosity of the firefighting foam under constant shear conditions ranging from 0.125 to 8 s^−1^. Similar to other aqueous foams, the firefighting foam also shows significant shear thinning; that is, the apparent viscosity decreases with the increasing shear rate. Under a constant shear rate, the stress response of the firefighting foam first decreases and then increases over time, and the lowest points of the curve cluster occur at *t* = 6 min. This nonmonotonic change is the same as the nonmonotonic effect of foam aging on the nonlinear region shown in Figure 6. By extracting the corresponding shear stress under different shear rates at a given time, as shown in Figure 14a, the flow curve of the firefighting foam under different aging times can be obtained. As shown in Figure 15, regardless of the aging time, the flow curves of the firefighting foam conform to the Herschel–Bulkley model shown in Equation (6). The fitting results of the Herschel–Bulkley model shown in Figure 16 reveal that the dynamic yield stress and flow behavior index both increase with the increasing aging time and that the consistency factor decreases, indicating that the firefighting foam becomes more plastic and shear-thinning.

(6) $$\tau =\tau_{y}+K{\dot{\gamma }}^{n}$$

### 3.5. Competitive Effects of Foam Drainage and Coarsening on Foam Rheology

Foam drainage and coarsening are the main manifestations of foam aging, which are coupled with each other and play different roles in affecting foam rheology. The decrease in the liquid fraction caused by foam drainage makes the foam stiffer, and the shear modulus increases. The increase in bubble polydispersity and average size caused by foam coarsening results in a decrease in shear modulus. Numerous experimental studies [33,34] and quantitative analyses, such as Equations (2) and (4), have shown the competitive effects of foam drainage and coarsening on the rheological response. Figure 6, Figure 7, and Figure 9 all show that the rheological parameters in the linear viscoelastic region change monotonically with the aging time, and the monotonic decline in the shear modulus indicates that the influence of foam coarsening on the rheological properties in this region is dominant. The variation in yield stress with the aging time almost monotonically increases, as shown in Figure 11, indicating that the influence of foam drainage on rheology appears to be dominant in the transition region. Both oscillatory shear in the nonlinear viscoelastic region (Figure 6) and viscous flow under slow shear (Figure 14) show nonmonotonic changes in rheological response with aging time, which are ultimately caused by the competition between foam drainage and coarsening.

### 3.6. Limitations and Prospects

Experimental investigations were conducted on the viscoelasticity, yielding, and viscous flow under slow shear of 3% AFFF/AR foam, along with analysis of competitive effects of drainage and coarsening on its rheological behavior. The selection of the 3% AFFF/AR foam as a starting point stems from its composition, which features the most complex surfactant–polysaccharide system among traditional non-fluorinated foam concentrates. Limited data indicate that classical rheological relationships for aqueous foams do not fully apply to 3% AFFF/AR foam. This discrepancy may arise from multiple factors, including the intrinsic complexity of the material and limitations in measurement techniques. As the foam ages, the local liquid fraction and bubble size exhibit top-down inhomogeneity within the foam container. However, rheological measurements inherently capture the average response within a specific region, whereas the local liquid fraction and bubble size are point-specific parameters. The combined effect of regional inhomogeneity and the relationship between local and average values may cause deviations in the constant factors of Equations (2) and (4). Alternatively, the intricate surfactant–polysaccharide composition unique to firefighting foams could be responsible. Furthermore, the qualitative conclusions from this study—such as the time-dependent decay of the local liquid fraction, time-dependent bubble growth, and the competitive influence of drainage and coarsening on rheology—are broadly applicable to other firefighting foams (e.g., fluoroprotein foams, fluorine-free foams). Nevertheless, quantitative differences are expected due to variations in foam formulation chemistry.

While this study and subsequent research on rheological behavior and structural aging focus on fundamental investigations into firefighting foams, foam fire-extinguishing numerical simulation serves as a critical conduit for applying these findings to practical fire suppression scenarios. Once the rheological properties of firefighting foams—and their variations with intrinsic factors (e.g., foam type, surfactants) and extrinsic factors (e.g., foam expansion ratio, thermal exposure)—are quantitatively characterized, they can be integrated into the governing equations of foam spread models. This integration enables the assessment of how rheologically induced changes impact real-world fire suppression, thereby establishing a quantitative framework for optimizing foam formulations. It should be noted, however, that achieving this objective requires extensive additional research.

## 4. Conclusions

Firefighting foam shows elasto-viscoplastic behavior. Under the oscillatory shear strain amplitude sweep mode, the firefighting foam passes through the linear viscoelastic region, transition region, and nonlinear viscoelastic region in sequence. The creep and relaxation response of firefighting foam in the linear viscoelastic region conforms to the Burgers model. In the transition zone, the foam yields are ductile and different from the classic brittle fracture yield. After entering the nonlinear viscoelastic region, the shear modulus of the firefighting foam changes significantly, and viscous deformation dominates the flow. The flow curve of the firefighting foam under slow shear can be effectively represented by the Herschel–Bulkley model.

Firefighting foam shows remarkable foam drainage and foam coarsening, which play different roles in affecting the foam rheology. Once the firefighting foam is prepared, the foam at different vertical positions continues to drain, becomes drier, the local liquid fraction decreases and gradually develops toward the equilibrium profile. Foam coarsening makes the bubbles more polyhedral, more dispersed, and larger in average size. Foam coarsening dominates the rheological response in the linear viscoelastic region, whereas foam drainage seems to dominate the foam yield change in the transition region. In the nonlinear viscoelastic region or slow shear flow, the competitive effects of foam drainage and coarsening are intense.

Although the experimental samples and data in this work are limited, some existing classical relationships among the rheological parameters, liquid fraction, and bubble size established for aqueous foam are not applicable to firefighting foam. To develop rheological relationships and constitutive models suitable for firefighting foam, a large amount of rheological test data must be accumulated. The present work provides basic protocols for measuring the rheology, drainage, and coarsening of firefighting foam. The effects of potential intrinsic factors—such as concentrate formulation, additive systems, and fluorine substitution—as well as extrinsic factors like expansion ratio and thermal exposure on firefighting foam rheology and aging will be gradually investigated in future work.

## Figures and Tables

**Table 1:** Mean bubble sizes of firefighting foam at specific time points and vertical positions, including arithmetic mean radius *R*_A_ (±standard deviation) and Sauter mean radius *R*_32_ (±bootstrap-estimated standard errors).

*t*/min	*R*_A_/mm					*R*_32_/mm				
	*z* = 3 cm	*z* = 5 cm	*z* = 7 cm	*z* = 9 cm	*z* = 11 cm	*z* = 3 cm	*z* = 5 cm	*z* = 7 cm	*z* = 9 cm	*z* = 11 cm
0.5	0.0502 ± 0.0213	0.0511 ± 0.0243	0.0495 ± 0.0257	0.0543 ± 0.0250	0.0540 ± 0.0253	0.0662 ± 0.0006	0.0714 ± 0.0007	0.0730 ± 0.0007	0.0745 ± 0.0007	0.0749 ± 0.0008
4	0.0703 ± 0.0339	0.0734 ± 0.0345	0.0795 ± 0.0353	0.0793 ± 0.0361	0.0896 ± 0.0414	0.0992 ± 0.0016	0.1020 ± 0.0016	0.1071 ± 0.0016	0.1073 ± 0.0015	0.1224 ± 0.0020
8	0.0810 ± 0.0437	0.0854 ± 0.0453	0.0958 ± 0.0499	0.1031 ± 0.0552	0.1312 ± 0.0670	0.1226 ± 0.0028	0.1279 ± 0.0027	0.1392 ± 0.0025	0.1527 ± 0.0030	0.1876 ± 0.0043
12	0.0873 ± 0.0521	0.1025 ± 0.0582	0.1168 ± 0.0665	0.1585 ± 0.0668	0.1939 ± 0.0790	0.1416 ± 0.0039	0.1583 ± 0.0037	0.1789 ± 0.0039	0.2065 ± 0.0050	0.2487 ± 0.0067
16	0.0985 ± 0.0634	0.1382 ± 0.0768	0.1586 ± 0.0847	0.1989 ± 0.1003	0.2417 ± 0.1064	0.1697 ± 0.0053	0.2091 ± 0.0057	0.2296 ± 0.0059	0.2783 ± 0.0075	0.3197 ± 0.0104
20	0.1099 ± 0.0774	0.1864 ± 0.0830	0.2215 ± 0.1073	0.2493 ± 0.1055	0.3036 ± 0.1220	0.2065 ± 0.0110	0.2516 ± 0.0088	0.2920 ± 0.0119	0.3251 ± 0.0122	0.3877 ± 0.0152

**Table 2:** Fitted parameters of the Burgers model for creep curves with 95% confidence intervals and goodness-of-fit metrics (RMSE).

*t*_hold_/min	*G*_1_/Pa	*μ*_1_/Pa·s	*G*_2_/Pa	*μ*_2_/Pa·s	RMSE
0.5	9.6 ± 0.2	62.7 ± 0.1	23.8 ± 1.1	327.8 ± 39.4	0.0015
4	6.7 ± 0.1	69.5 ± 0.2	10.8 ± 0.4	225.1 ± 13.6	0.0018
8	6.2 ± 0.1	84.1 ± 0.2	8.3 ± 0.2	152.4 ± 5.8	0.0015
12	5.9 ± 0.1	115.2 ± 0.7	4.5 ± 0.1	124.0 ± 2.7	0.0017
16	5.4 ± 0.1	142.3 ± 1.4	3.9 ± 0.1	109.4 ± 2.5	0.0021
20	4.9 ± 0.1	173.9 ± 3.8	3.2 ± 0.1	98.0 ± 3.0	0.0034

## Data Availability

The original contributions presented in this study are included in the article. Further inquiries can be directed to the corresponding author(s).

## Abbreviations

The following abbreviations are used in this manuscript:

Nomenclature	
*a*, *b*, *c*	power law exponents
*a*_1_, *a*_2_	empirical parameters in Equation (4) and Equation (5)
*d*_Fe_	Feret diameter [mm]
*f*	frequency [Hz]
*G*′	storage modulus [Pa]
*G*″	loss modulus [Pa]
*G*_1_, *G*_2_	parameters of the Burgers model (Equation (3)) [Pa]
*J*	compliance [Pa^−1^]
*J*∞	residual compliance [Pa^−1^]
*K*	consistency factor [Pa.s*^n^*]
*n*	flow behavior index
*r*	bubble radius, equal to half the Feret diameter [mm]
*R*_32_	Sauter mean radius, defined as *R*_32_ = <*r*^3^>/<*r*^2^> [mm]
*R*_A_	arithmetic mean radius, defined as *R*_A_ = <*r*> [mm]
*R*_o_	cubic mean radius, defined as *R*_o_ = <*r*^3^>^1/3^ [mm]
*R*_rms_	mean square radius, defined as *R*_rms_ = <*r*^2^>^1/2^ [mm]
*t*	time [min]
*t*_hold_	holding time [min]
Greek symbols	
*γ*	strain
*γ*_0_	strain amplitude
*γ*_y_	yield strain
$\dot{\gamma }$	shear rate [s^−1^]
*δ*	loss angle [°]
*μ*_1_, *μ*_2_	parameters of the Burgers model (Equation (3)) [Pa.s]
*σ*	surface tension of the foam solution [mN.m^−1^]
*τ*	shear stress [Pa]
*τ*_0_	stress amplitude [Pa]
*τ*_y_	yield stress [Pa]
*ϕ_l_*	liquid friction (volume fraction of the liquid phase of a foam)
$\phi_{l}^{*}$	critical liquid fraction
*ϕ_l_*_,0_	initial liquid friction
*ψ*	relative conductivity of firefighting foam, defined as *ψ* = *ψ*_foam_/*ψ*_solution_
*ψ*_foam_	conductivity of firefighting foam [mS.cm^−1^]
*ψ*_solution_	conductivity of the foam solution [mS.cm^−1^]
